# Molecular docking-based screening of methicillin-resistant *Staphylococcus aureus* FEM proteins with FDA-approved drugs

**DOI:** 10.6026/973206300191035

**Published:** 2023-11-30

**Authors:** Anjini Gayatri Akkiraju, Aishwaraya Badugu, Aditi Das, Someswar Rao Sagurthi

**Affiliations:** 1 Molecular Medicine Lab, Department of Genetics & Biotechnology, Osmania University, Hyderabad, Telangana, 500007, India

**Keywords:** Pentaglycine, antimicrobial resistance, inhibition, docking, virtual screening and FEM proteins

## Abstract

Antibiotic resistance stands as one of the most significant public health challenges in recent decades. FEM proteins are responsible for the synthesis of pentaglycine cross-bridge, a primary constituent of bacterial peptidoglycan polymer crosslinking during cell wall biosynthesis. Since they are necessary for bacterial survival and antibiotic resistance, they were considered as significant antibacterial targets. We report herein, the virtual screening and selection of FDA-approved drugs and their potent similar molecules as FEM protein inhibitors and analyzed for inhibiting affinity and their ADMET pharmacokinetic properties. This data provide a foundation for the development and optimization of structurally innovative antimicrobial drugs.

## Background:

*Staphylococcus aureus* causes a wide spectrum of common ailments that can be acquired from the community and in hospitals. Due to lower responsiveness to commercially available drugs, the rise of Methicillin-resistant *Staphylococcus aureus* (MRSA) clinical isolates has increased and evolved into various multi-drug resistant strains making the treatment difficult. The foreign expression of altered PBP2a performs the host PBP function and governs the strategy of β-lactam antibiotic resistance in methicillin-resistant *S. aureus* (MRSA) [1]. Therefore, the identification of new targets and effective drug synthesis is necessitated to overcome the MRSA which is now poses a global health concern. A thorough understanding of the mechanisms behind methicillin resistance opened the door to the identification of several targets like FEM, which contributes to the *S. aureus* methicillin resistance [2]. Even though there are numerous variables involved in the development of multidrug resistance in MRSA, the proteins of the *femABX* family are one of the key elements required for resistance. During the formation of the bacterial cell wall, these non-ribosomal peptidyl transferases are in charge of catalyzing the transfer of glycine residues to the developing interpeptide. Notably, FEM proteins were recognized as strict in the substrate specificity, thus serving as a platform for numerous cell wall-sorted attachments with pathogenic proteins and are necessary for *S. aureus* viability [3, 4]. Peptidoglycan is a fundamental component required for microbial cell growth, division, and maintenance of structural integrity. It uses several repeated units of N-acetylmuramic acid (MurNAc) and N-acetylglucosamine (GlcNAc) macromolecules interlinked via pentapeptide stem (5-Glycine) as a backbone to build bacterial cell wall biosynthesis and signifies as an important target for novel drug development [5]. Three constitutive FEM proteins namely, FemA, FemB, and FemX are the family of transpeptidase proteins encoded by the *femABX* operon and are responsible for the formation of muropeptide pentaglycine bridge formation. This regulates the main building blocks called peptidoglycan polymer cross-linking [6]. These FEM proteins were essential for bacterial survival and to date, no human homologs were identified. Thus, inhibition of these pentaglycine bridge-forming FEM proteins serves as a promising approach to combat bacterial resistance.

## Materials and methods:

## Homology model building of FemB and *insilico* evaluation of model accuracy:

The structural characterization of *S. aureus* FemX and FemC has been reported earlier with crystal structures and were obtained from the RCSB PDB protein data bank (www.rcsb.org) with 1.62Å and 2.35Å resolution (PDB ID: 6SNR and 7TEA respectively) [7, 8]. FemB protein template was retrieved from the UniProt database (UniProt ID: P0A0A6.FEMB_STAAM) and confirmed with BLASTp, performed homology modeling using a server ITASSER, to obtain protein 3D structure [9]. This server generated five homology models and all of them were further subjected to SAVES 6.0 and RAMPAGE validations to determine the top best model with improved properties of stereochemistry, and quality [10].

## Active site prediction:

Ligand-binding active sites were not well characterized for all FEM proteins. Hence, we evaluated FEM protein 3D structures under CASTp to identify potential ligand binding sites/pockets (active sites) and the cavities which were further validated through SCFBio and Depth active site prediction web servers [11]. To forecast and define possible binding pockets in the freshly revealed 6SNR and 7TEA crystals, we have used the web server application called Do G Site Scorer [12,13, 14]. It assists in locating possible binding pockets inside a given protein and ranks the accessible pockets based on their size, surface, and drug ability scores.

## Structure-based virtual-screening of ligands:

The ZINC15 and Drug Bank databases were used for the extraction of 1,600 FDA-approved small-molecule medicines in .SDF format [15, 16]. After that, some of the compounds with molecular weights of more than 500 were eliminated. The 1,530 molecules with 3D structures were used for molecular docking. For virtual screening, the ligands were translated into .mol format with the use of the Open Babel program [17] and added hydrogen atoms, cleaned in 2D and 3D, and prepared in .PDB formats for docking studies by using MarvinSketch 5.6.0.2. and its allied applications (1998-2011, Copyright © ChemAxon Ltd).

## Caver Web-mediated virtual screening of ligands specific to FemB protein:

Caver Web is a fully automated preset program that involves fast and effective screening of FDA-approved drugs from the ZINC15 database. It is a computerized program that runs with a single click to start and scan the approved drugs for fitting into protein PDB [18]. It employs a hybrid technique that combines molecular docking with ligand transport studies over a broad spectrum of legal medications. The results obtained after submitting the job generate a plot graph for each interacting molecule and archive all data together in a summary .excel form to retrieve the ligand binding features. It displays the transport binding energy profile for every participated drug in a single plot and simplifies the comparison analysis step to find the potent interactive molecules. This makes it as an easily available free service to access the broad spectrum of the scientific community. The regular complexity achieved during the evaluation of huge library input was avoided with this comprehensive, time-effective CaverDock calculation. The initial step involves a rapid evaluation of ligand binding energy in the active site using the fast AutoDock Vina algorithm. The second step leads to identifying the potent top 50 binders based on their predicted bound energies using the CaverDock algorithm. The discretization parameters were set as default to define the distance between the centers of the tunnel. Standard caver settings were used in the study with 0.9 minimum probe radius, 4 and 3 as shell depth, radius respectively for maximal depth of a protein surface region, and 3.5 clustering threshold with a maximal distance of 3 and 5 as the desired radius. Using the binding energy as default quality control, the CaverDock result displayed minimal energy and involved bottleneck residues of the lower-bound trajectory.

## Molecular docking:

Virtual screening of protein-ligand interaction at the molecular level was performed using the Molegro Virtual Docker (MVD 2012.5.5) [19]. To start with this, a receptor grid was generated in the docking wizard, and the receptor molecule and ligand were prepared by following the manual instructions. FEM proteins were prepared in docking space by assigning charges, missing bonds, and fit bond orders at required spaces and the final construction was preserved in .pdb format. Simultaneously, the molecule library was cleaned and uploaded into the docking tool wizard for further optimizations for screening. This molecular docking result reveals the molecular information regarding the ligand's interactions with the target protein. The protein-ligand complex interactions were represented through a mathematical notation called Moldock score, which is based on Piecewise Linear Potential (PLP). The overall Moldock score is comprised by adding the whole internal ligand and protein interaction energies, and its soft penalties. The ligand complex with the greatest moldock score therefore displays the highest binding energies with favorable ligand-protein interactions. The lead-hit drug molecules based on binding affinity and H-bond scores were further analyzed for active site interactions through the PyMol and Biovia Discovery Studio 2020 Client.

## ADMET profiling of FDA-approved hit similar molecules:

The best docked ligand entities were selected and characterized for pharmacokinetic property predictions using one step pkCSM online freely available webserver. It provides a precise and thorough *in silico* ligand ADMET properties. It involves the use of molecule in SMILES file as input source and internally calculates all properties in shortest and distant paths, finally predicts ADMET properties and results in the output file with the list of features with the molecular structure of the ligand.

## Result and discussion:

FEM proteins which play a key role in the biosynthesis of *Staphylococcus aureus* cell wall barrier were recently characterized and their structural features were identified to act as a molecular target for MRSA inhibitions. Blockade or inhibiting the FEM protein by the interaction of the ligand with active residue alleviates the MRSA infectious phenotype and aims to raise the susceptibility with existing antibiotics on combinatorial approach. This suggests the FEM proteins as a potential target approach to kill MRSA. Virtual screening of FDA-approved drugs against the ligand-binding domain of FEM proteins was carried out by molecular docking analysis and ligands with the highest potency towards FEM protein was listed through Caver Web. Drugs similar with the highest binding than hit molecules with minimum binding scores were examined to act as potential MRSA FEM inhibitors. Biovia Discovery Studio revealed 3D, 2D-complex structures that have displayed strong interactions to find the active residue participations. These employed accurate computational results might have a fast-track approach to identify, and develop novel treatments against MRSA. The FDA-approved medications covered in this study can be used together for combinational therapies, which work against MRSA resistance and antibiotic sensitivity.

## FEM proteins structure prediction and ligand preparation:

The FemB homology model obtained from ALPHAFOLD has yielded a quality of 96.5% accuracy, with 100% favorable ERRAT results for the pattern of non-bonded atomic interactions. A total of 93% of the residues have averaged 3D-1D score >= 0.1 and are termed to be qualified (As per the principle of ERRAT results at least 80% of the amino acids have scored >= 0.1 in the 3D/1D profile). The RAMPAGE plot with 118 structure analysis at 2.0A° resolution, R-factor no > 20%. Among the total of 419 amino acid residues, 96.6% are the most favored, with 2.9% allowed and 0.5% disallowed (Figure S1). In the process of potent inhibitor identification against FemC and FemX proteins, we have obtained the crystal structure in .pdb format from RCSB PDB source with ID: 7TEA and 6SNR, respectively.

## FEM protein active site predictions:

*Staphylococcus aureus* FEM proteins were not well established for active drug interaction sites. Given this, an initial guess for active site amino acid residues on each FEM protein was determined by using the PDB BLAST web server. It defined the possible alignment of efficient ligand interactive residues in proteins via comparative studies. Later, CASTp 3.0 and depth validation web services discovered the potential ligand binding active site residues on FEM protein 3D structures (http://sts.bioe.uic.edu/castp/). The employed DoGSiteScorer grid-based webserver tool has identified the potential binding pockets in each FEM model. The crystal PDB introduced in the workspace was analyzed for active site prediction and identified the depth region possible binding sites, and volume. Top hit pockets were selected and listed (Table S1) by ranking from size, surface area involved, and druggability scores. This screening plays a key role in parameter settings for the preferred protein-ligand molecular dockings. In the current investigation, the identified top hit pocket position (P1) served as the coordinate source for our protein grid arrangement and subsequent docking analysis.

## CaverDock screening of FDA-approved drugs against *S. aureus* Fem B:

**Figure 1:**
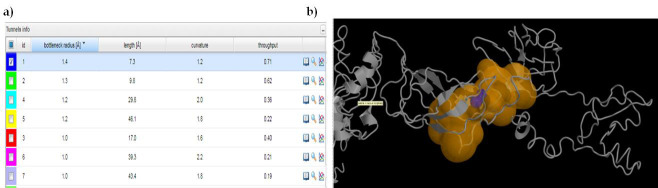
Tunnel visualization for FemB pocket with a list of bottleneck descriptions

**Figure 2:**
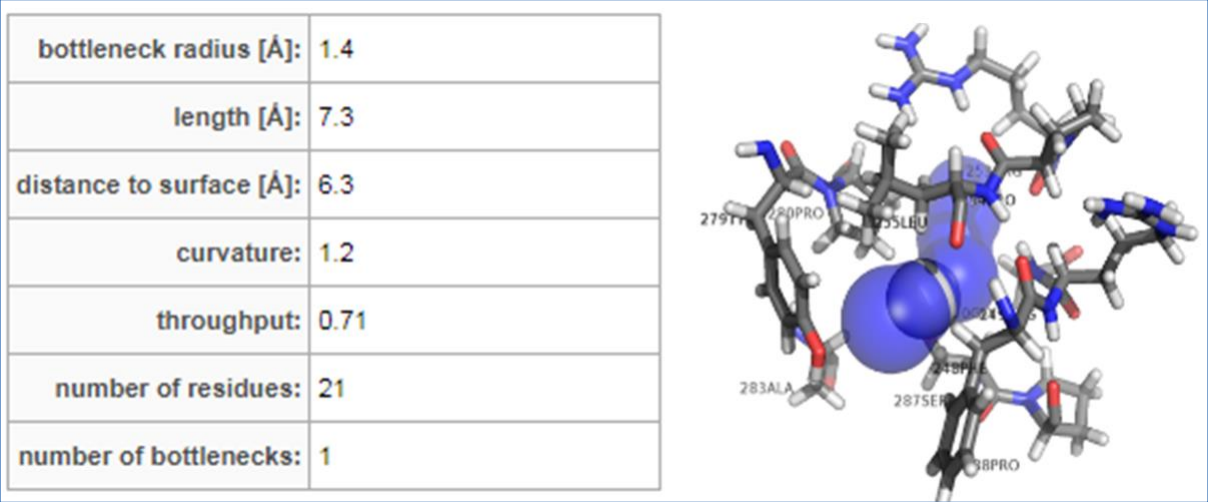
3D overview and list of bottleneck residues involved in top-hit FemB tunnel

There was no available literature confirming the active residues for FemB drug designing therefore we used Caver server for active site residue characterization. To achieve this, the FemB homology model is.PDB format is used as an input source for the Caver web computations (https://loschmidt.chemi.muni.cz/caverweb). It identified the binding residual data regarding the protein tunnels, which helps in the transportation of small molecule ligands into the active site of the enzymes. A total of 15 relevant tunnels were initially detected and all of them were opened by volume more than 1 Å Figure 1a. A fine hit tunnel which proposes to be a potent biologically relevant tunnel was selected based on the high relevance score and opted to screen FDA-approved drugs for selection of repurposing pharmaceuticals Figure 1b. An overview of the selected tunnel with data descriptions was displayed in Figure 2 and all tunnel sets were listed in Table 1 accordingly. Further, ligands were screened from the ZINC database through Caver docking calculations. After docking highest scored and better interacting leads were selected.

Leads were selected by considering the parameters like good binding energy towards FEM proteins by following the principle of linear correlation. However, the other tunnels were also grouped under the well-acceptable main tunnel, but a slight modification is only with a difference in opening size on the protein's surface and its significant energy barrier involved in the ligand transport.

## Structure-based virtual screening of FDA-approved drugs:

The downloaded FDA-approved drugs comprise several diverse molecules such as synthetic and natural sources namely peptide-based drugs, anti-HIV, anti-malarial, anti-Hepatitis C virus, antibacterial, antifungal, and anti-inflammatory. Using default parameters, unless mentioned separately, the molecules which are customized to focus as antibacterial agents were listed and screened for docking against prepared Fem X, C crystals PDB's and FemB homology model. This docking gave an output of binding energy, molecular interactions with hydrogen bondings and torsions, rerank scores. The molecular ranking was prepared based on their best binding energy and maximum hydrogen bonding affinity towards active site residues of FEM proteins. Among all FDA-approved drugs, the following listed molecules were identified as hit molecules against FEM proteins. Lumacaftor (Pubchem ID: 90080139), Olaparib (Pubchem ID: 155309251), and Dihydroergocornine (Pubchem ID: 11870334) against FemX and Glycocholic acid (Pubchem ID: 10140), Hupehenine (Pubchem ID: 14240934) and Limonin (Pubchem ID: 179651) against FemC protein.

To date no inhibitors were reported against FemB, so FDA-approved ZINC database screening was performed using the Caver Web server, which revealed a list of molecules against FemB namely, 3-Hydroxyhippurate (Pubchem ID: 6931069), Procodazole (Pubchem ID: 65708) and Succinanilic acid (Pubchem ID: 7598). Initially, all individual molecules from the ZINC15 database were evaluated in the process of searching for the potent FemB inhibitor (Figure S2a). Based on the results from best-scoring, binding energies, ZINC6534965 (3-Hydroxyhippurate), and ZINC51581 (Procodazole) were selected (Figure S2b). Based on this demonstrated report as a query ligand source, a similarity search (Tanimoto fingerprint with 95% similarity) was performed against the PubChem database, and found a good number of similar molecules with potent FEM protein interactions to act as potent FEM inhibitors. The shortlisted potent FDA-approved drug similar molecules with the best binding energy were listed in the following Table 2, Table S2 according to their moldock score; rerank score and active site interactions. Therefore, mentioned molecular docking pictures have shown the predominance in the association of various pi, hydrogen bond interactions amongst ligands, and FEM protein amino acid residues (Figure S3, Table 2). Further screening and analog diversification are required in chemical synthesis to progress these molecules as FEM inhibitors. These results helped in the identification of potential FDA-approved drugs and could be used for repurposing against FEM proteins to inhibit the MRSA pathogenicity. Conserved substrate-binding residues of *S. aureus* FEM proteins can further be denoted as active site residues to inhibit MRSA viability.

## Potent hit similar ligand identification and its interaction with FEM proteins:

Virtual screening has been carried out to discover the potent FDA-approved drugs which bound to the FEM protein active sites and their binding energies were predicted using MVD software molecular docking approaches. The potential hits (out of FDA-approved candidate library) were listed based on their scores and bound energies for the FEM proteins (Table 3).

**Figure 3:**
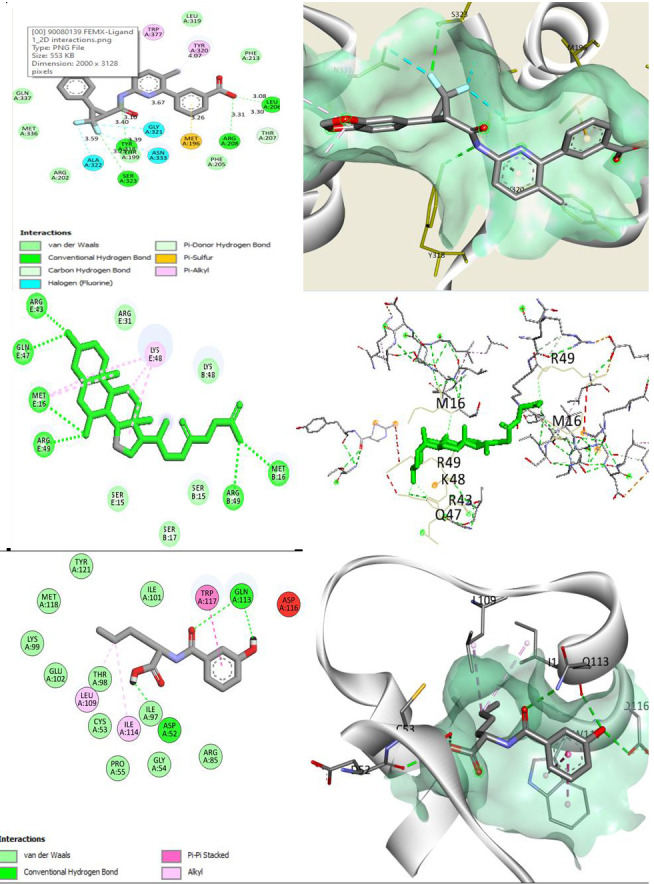
Two and three-dimensional interactions of FDA-hit similar against *S. aureus* FEM proteins.All ligand interaction patterns against FEM proteins were listed above showing the left panel with ligand-protein interaction in detail and the right panel with the 3D conformation of ligand binding. Ligands were displayed in stick models and figures were generated using Biovia Discovery Studio Imaging 2020 Client. 3a) Lumacaftor similar 3-[6-[[3-(1,3-Benzodioxol-5-yl)-2,2-difluorocyclopropanecarbonyl]amino]-3-methylpyridin-2-yl]benzoic acid (PubChem ID: 90080139) against FemX. 3b) Glycocholic acid similar (PMID: 90473176) against FemC. 3c) 3-Hydroxyhippurate similar namely (PMID: 150666978) against FemB.

In comparison to the standard FDA drugs, the similar molecules that possess the best docking score with drug-like effectiveness to the majority of compounds were predicted and analyzed. Docking performance has shown the Lumacaftor similar 3-[6-[[3-(1,3-Benzodioxol-5-yl)-2,2-difluorocyclopropanecarbonyl]amino] -3-methylpyridin-2-yl]benzoic acid (PubChem ID: 90080139) against FemX with highest binding energy of -137.66 among all by forming a Pi-sulfur and Pi-alkyl interaction with TYR320, 377 and MET196. The whole complex has involved many hydrogen bonding with several residues listed in active site amino acids of the FemX receptor (Figure 3a, Figure S3a). In addition, hydrophobic interactions were observed which further depicts the ability of ligands to bind to the active site of FemX and also stabilize the interaction between the receptor and ligand. Glycocholic acid similar (PMID: 90473176) has shown a binding energy of -110.09 where several alkyl bondings were seen with minor hydrogen bonds towards FemC (Figure 3b, Figure S3b). The 3-Hydroxyhippurate similar namely (PMID: 150666978) has interacted with FemB forming one Pi-Pi- stacked interaction and two alkyl bonds engaging with the TRP117 and LEU109, ILE114 residues respectively with binding energy of -84.67 (Figure 3c, Figure S3c). Apart from the scores, several hydrogen bonds were seen among the interactions, which might enforce the potential binding affinity towards the receptor.

## Predicted ADMET properties of selected molecules by pkCSM webserver:

An overview of selected chemical molecules and clinical features with pharmacokinetic properties were analyzed using the pkCSM webserver and labeled in Table S3, Table S4 and Table S5. Selected compounds showed acceptable ADMET properties and could be used for *invitro* evaluations. For the first time this manuscript reported the FemB inhibitors from FDA approved drug molecules, which can be possibly repurposed.

## Conclusion:

The limited therapeutic spectrum against MRSA necessitates an urgent development of antibiotics and medications to enhance the current antibiotic efficacy and drugs to enhance the existing antibiotic efficacy. We report the optimal binding features of structure-based FDA-approved drugs repurposed to treat MRSA pathogenic infections. It helps to cross the emergence by following certain time limit management in collaboration with certain existing *invitro* results. In this work, we virtually examined a library of FDA-authorized drugs from ZINC15 and DrugBank databases against *S. aureus* FEM proteins. Three lead molecules were shortlisted based on their docking scores and prioritized based on their minimum binding energy and active interactions with the FEM proteins. In this study, we also evaluated the molecules solubility and toxicity properties.

## Declaration of Interest:

The authors declare that they have no known competing interests (or) personal relationships that could have appeared to influence the work reported in this manuscript. The research leading to these results received funding from the DST-SERB-ECR grant under file no: ECR/2017/003381.

## Figures and Tables

**Table 1:** Caver web identified tunnel characteristics of *S.aureus* FemB protein

**Tunnel cluster**	**Throughput**	**Cost**	**Bottleneck radius**	**Bottleneck R error bound**	**Length**	**Curvature**
1	0.708293	0.344897	1.363934	-	7.289116	1.152357
2	0.621153	0.476178	1.317121	-	9.752019	1.220999
3	0.403553	0.907448	0.977079	-	17.0398	1.598636
4	0.356038	1.032718	1.198837	-	29.79473	2.004507
5	0.217235	1.526775	1.198837	-	46.08838	1.779228
6	0.205353	1.583026	0.995756	-	39.25812	2.165835
7	0.187225	1.675445	1.041309	-	43.388	1.768461
8	0.153954	1.871102	1.000415	-	48.89275	1.873098

**Table 2:** Virtual screening results of FDA-approved similar compounds against FEM proteins

**Ligand PubChem ID**	**MolDock Score**	**Re-rank Score**	**Interaction**	**Internal**	**Electro**	**H-Bond**
**Potent FDA-approved similar compounds against FemXprotein.**
Lumacaftor similar_90080139	-137.66	-107.41	-165.31	27.6504	0	-5.15
Olaparib similar_155309251	-131.07	-102.36	-159.57	28.5	0	-0.36
Dihydroergocornine similar_11870334	-164.3	-126.88	-167.35	3.05	0	-5
**Potent FDA-approved similar compounds against FemC protein.**
Glycocholic acid similar_90473176	-110.09	-78.61	-126.24	16.15	0	-18.16
Hupehenine similar_122173174	-85.52	-66.16	-95.82	10.29	0	-8.21
Limonin similar_95223056	-78.96	-49.67	-81	2.09	0	-12.76
**Potent FDA-approved similar compounds against FemB protein.**
3-Hydroxyhippurate similar_150666978	-84.67	-68.46	-91.57	6.9	0	-7.07
Procodazole similar_163586828	-87.83	-67.32	-88.08	0.25	0	-3.6

**Table 3:** List of proposed FDA-approved drugs as repurposed drugs and their screened structure similar molecules which possess higher binding affinity against *S. aureus* FEM proteins.

**Sl.no**	**Protein**	**PubChem ID**	**Parent Compound**	**Similar compounds (90%)**
1	**Fem X**	90080139	Lumacaftor	165
		155309251	Dihydroergocornine	407
		11870334	Olaparib	116
2	**Fem B**	6931069	3-Hydroxyhippurate	860
		65708	Procodazole	675
		7598	Succinanilic acid	270
3	**Fem C**	10140	Glycocholic acid	862
		14240934	Hupehenine	199
		179651	Limonin	327
