# Supplementary material for: Molecular docking-based screening of methicillin-resistant Staphylococcus aureus FEM proteins with FDA-approved drugs
Source: Bioinformation. 2023 Nov 30;19(11):1035–42. doi: 10.6026/973206300191035 (PMC10692981; doi:10.6026/973206300191035)
Supplement: Data 1 [file 973206300191035S1.pdf]

Supplementary material

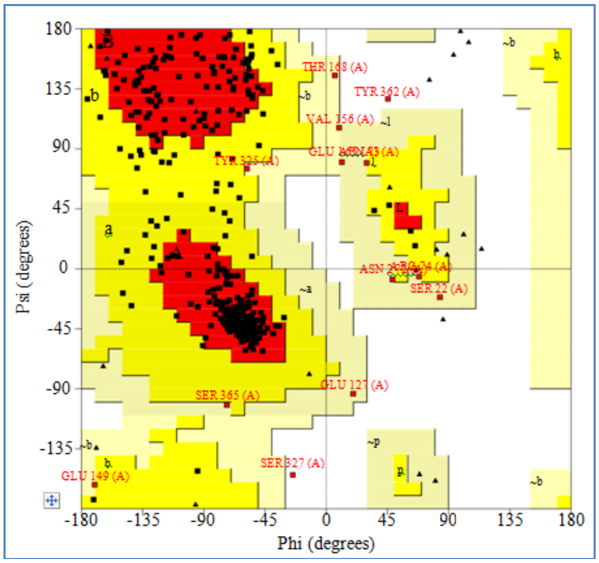

Figure S1: RAMPAGE plot statistics for FemB model

a) b)

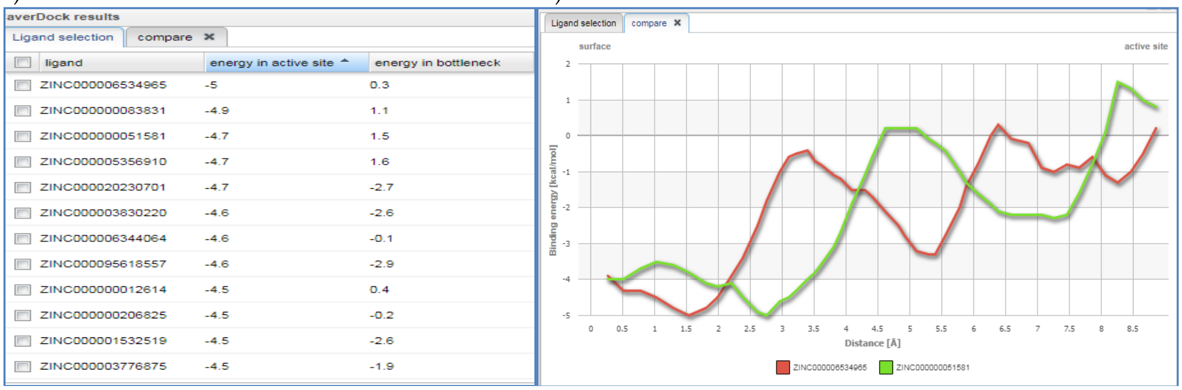

averDock results

ligand	energy in active site	energy in bottleneck
ZINC000006534965	-5	0.3
ZINC000000083831	-4.9	1.1
ZINC000000051581	-4.7	1.5
ZINC000005356910	-4.7	1.6
ZINC000020230701	-4.7	-2.7
ZINC000003830220	-4.6	-2.6
ZINC000006344064	-4.6	-0.1
ZINC000095618557	-4.6	-2.9
ZINC000000012614	-4.5	0.4
ZINC000000206825	-4.5	-0.2
ZINC000001532519	-4.5	-2.6
ZINC000003776875	-4.5	-1.9

Figure S2: Caver web docking results. a) Caver docking results for hit binding molecules and b) Comparison between top scored ligands for active site binding, energy.

Table S1. DoGSite Scorer descriptors of *S. aureus* FEM X, C and B protein model binding pockets.

Pocket No.	Volume (Å³)	Surface area (Å²)	Depth [Å]
**FemB-P1**	658.43	1650.58	28.28
**FemB-P2**	573.95	1468.99	23.70
**FemX-P1**	669.7	1412.62	29.03
**FemX-P2**	156.67	614.42	15.37
**FemC-P1**	285.18	777.00	28.82
**FemC-P2**	263.68	803.72	22.92

Table S2: Estimated bound free energies forFemB with potential hit repurposed drugs.

FDA approved drug	Pubchem ID	MolDock Score	Rerank Score	H-Bond
**3-Hydroxyhippurate**	6931069	-137.66	-107.41	-5.15
**Procodazole**	65708	-131.07	-102.36	-0.36
**Succinanilic acid**	7598	-164.30	-126.88	-5.00

a) 2D, 3D interactions of hit molecules against with FemX

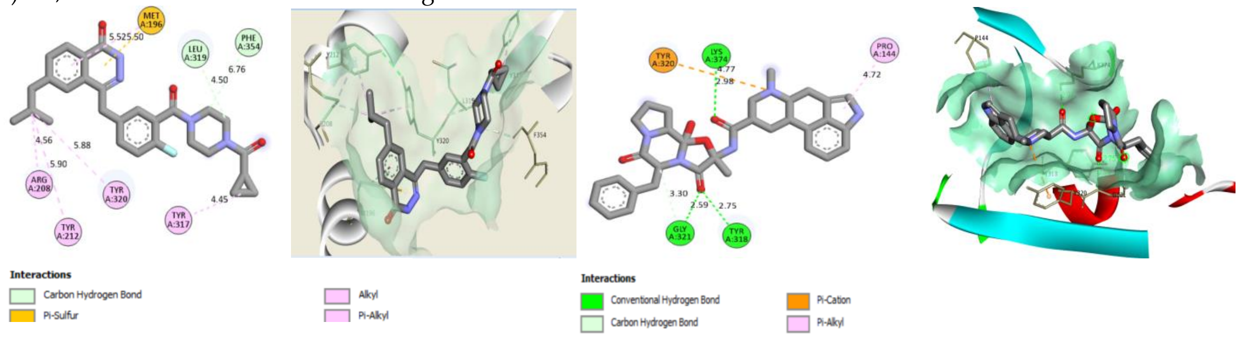

b) 2D, 3D interactions of hit molecules against with FemC.

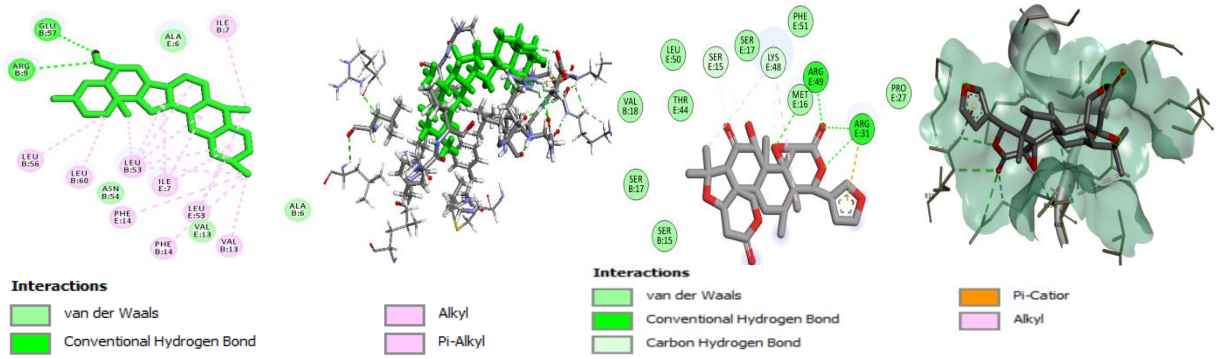

c) 2D, 3D interactions of hit molecules against with FemB.

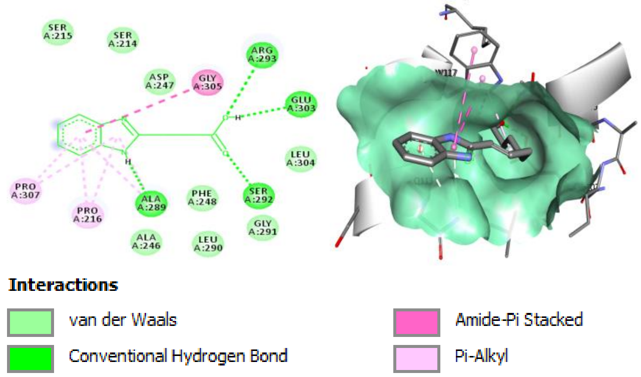

Figure S3: 2D, 3D dimensional interactions of FDA-hit similars against *S.aureus* Fem proteins.

Table S3. Details of the final identified hit compounds

S. No.	Compound pub chem ID	Formula	SMILES
**1**	90080139	$C_{24}H_{18}F_2N_2O_5$	CC1=C(N=C(C=C1)NC(=O)C2C(C2(F)F)C3=CC4=C(C=C3)OCO4)C5=CC(=CC=C5)C(=O)O
**2**	155309251	$C_{28}H_{31}FN_4O_3$	CC(C)CC1=CC2=C(C=C1)C(=O)NN=C2CC3=CC(=C(C=C3)F)C(=O)N4CCN(CC4)C(=O)C5CC5
**3**	11870334	$C_{33}H_{38}N_5O_5^+$	CC1(C(=O)N2C(C(=O)N3CCCC3C2(O1)O)CC4=CC=CC=C4)NC(=O)C5CC6C(CC7=CNC8=CC=CC6=C78)[NH+](C5)C
**4**	90473176	$C_{26}H_{43}NO_6$	CC(CCC(=O)NCC(=O)O)C1CCC2C1(C(CC3C2C(CC4C3(CCC(C4)O)C)O)O)C
**5**	122173174	$C_{27}H_{45}NO_3$	CC1CCC2C(C3CCC4C(C3CN2C1)CC5C4CC(C6C5(CCC(C6)O)C)O)(C)O
**6**	95223056	$C_{28}H_{32}O_{10}$	CC(=O)OC1C2C(OC3C2(COC(=O)C3)C4CCC5(C(OC(=O)C6C5(C4(C1=O)C)O6)C7=COC=C7)C)(C)C
**7**	150666978	$C_9H_8BrNO_4$	C1=CC(=CC(=C1)O)C(=O)NC(C(=O)O)Br
**8**	163586828	$C_{10}H_9N_3O_2$	C1=CC=C2C(=C1)NC(=N2)CC(=N)C(=O)O

Table S4: Pharmacokinetic properties of potent hit molecules predicted by pkCSM webserver

S. No.	Protein target	Compound pubchem ID	Molecular Weight (Da)	Log P	Rotatable Bonds	H-bond Acceptors	H-bond Donors
**1**	FemX	90080139	452.4	4.47	5	5	2
**2**		155309251	490.5	3.54	6	4	1
**3**		11870334	584.6	0.66	4	5	4
**4**	FemC	90473176	465.6	2.56	6	5	5
**5**		122173174	431.6	3.67	0	4	3
**6**		95223056	528.5	2.67	2	10	0
**7**	FemB	150666978	274.0	0.92	3	3	3
**8**		163586828	203.2	1.20	3	3	3

Table S5: ADMET (absorption, distribution, metabolism, excretion, and toxicity) properties of hit similars predicted by pkCSM webserver

Properties (Units)	90080139	155309251	11870334	90473176	122173174	95223056	150666978	163586828
**Absorption**								
**Water solubility (log mol/L)**	-3.16	-4.30	-2.93	-4.03	-3.92	-3.99	-2.37	-2.86
**$Caco_2$ permeability (log Papp in 10-6 cm/s)**	0.73	1.00	0.21	0.45	0.84	1.09	0.71	0.63
**Intestinal absorption (human) (%)**	74.52	93.71	57.76	44.63	92.73	100	76.52	83.02
**Skin Permeability (log Kp)**	-2.73	-2.73	-2.73	-2.73	-3.46	-2.74	-2.73	-2.73
**P-glycoprotein substrate**	Yes	Yes	Yes	Yes	Yes	No	No	Yes
**P-glycoprotein I inhibitor**	No	Yes	Yes	No	Yes	Yes	No	No
**P-glycoprotein II inhibitor**	No	Yes	Yes	No	No	No	No	No
**Distribution**								
**VDss (human) (log L/kg)**	-1.69	-0.30	1.32	-1.08	0.11	0.22	-0.91	-0.39
**Fraction unbound (human) (Fu)**	0.02	0	0.30	0.26	0.32	0.11	0.64	0.28
**Blood Brain Barrier (BBB) Permeability (Log BB)**	-0.54	-0.79	-0.33	-0.81	-0.03	-1.25	-0.78	-0.89
**CNS permeability (Log PS)**	-2.86	-2.46	-2.98	-3.14	-1.92	-3.07	-3.00	-2.76
**Metabolism**								
**CYP2D6 substrate**	No	No	No	No	No	No	No	Yes
**CYP3A4 substrate**	No	Yes	Yes	Yes	Yes	Yes	No	No
**CYP1A2 inhibitor**	No	No	No	No	No	No	No	No
**CYP2C19 inhibitor**	No	Yes	No	No	No	No	No	No
**CYP2C9 inhibitor**	No	Yes	Yes	No	No	No	No	No
**CYP2D6 inhibitor**	No	No	No	No	Yes	No	No	No
**CYP3A4 inhibitor**	No	Yes	Yes	No	No	Yes	No	No
**Excretion**								
**Total Clearance (log ml/min/kg)**	-0.079	0.41	0.95	0.65	-0.08	-0.01	-0.007	0.79
**Renal OCT2 substrate**	No	No	No	No	No	No	No	No
**Toxicity**								
**AMES toxicity**	No	No	No	No	No	No	Yes	No
**Oral Rat Acute Toxicity (LD50) (mol/kg)**	2.79	2.51	2.71	2.07	2.78	3.82	2.50	2.39
**Oral Rat Chronic Toxicity (LOAEL) (log mg/kg_bw/day)**	1.52	2.11	3.22	2.53	1.26	1.36	1.87	1.90
**hERG I inhibitor**	No	No	No	No	No	No	No	No
**hERG II inhibitor**	No	Yes	Yes	No	Yes	No	No	No
**Skin Sensitisation**	No	No	No	No	No	No	No	No
**T.Pyriformis toxicity**	0.28	0.29	0.28	0.28	0.30	0.28	0.28	0.28
